# Editorial: Advanced research on abdominal and thoracic aortic aneurysms: new insights into molecular mechanisms

**DOI:** 10.3389/fcvm.2023.1304498

**Published:** 2023-10-19

**Authors:** Venkateswaran Subramanian, Haruhito Adam Uchida, Toru Miyoshi

**Affiliations:** ^1^ Department of Medicine—Cardiovascular Medicine, University of Missouri, Columbia, MO, United States; ^2^ Department of Chronic Kidney Disease and Cardiovascular Disease, Okayama University Faculty of Medicine, Dentistry and Pharmaceutical Sciences, Okayama, Japan; ^3^ Department of Cardiovascular Medicine, Okayama University Faculty of Medicine, Dentistry and Pharmaceutical Sciences, Okayama, Japan

**Keywords:** aortic aneruysm, abdominal aortic aneurysm, thoracic aortic aneurysm, diabetes, RNA seq

**Editorial on the Research Topic** Advanced research on abdominal and thoracic aortic aneurysms: new insights into molecular mechanisms

Abdominal and Thoracic aortic aneurysmal diseases are devastating with a high risk of rupture that frequently leads to death. Regardless of significant improvements in the comprehension of aortic aneurysmal disease development, a number of questions need to be addressed to clarify the conflicts in the research findings. This special research topic in Frontiers in Cardiovascular Medicine includes two original research articles, one review article, and one mini-review article aimed to highlight the molecular mechanisms of aortic aneurysms.

Abdominal aortic aneurysms (AAAs) are permanent dilations of the abdominal aorta and the most common aortic aneurysms in humans (1). In this special issue, in an original article, Ladd and colleagues utilized two different experimental models of AAAs—a well-established topical elastase treatment model of AAA and a more rupture-prone combination model of elastase and lysyl oxidase inhibitor, beta-aminopropionitrile (BAPN) and provided solid data that pharmacological inhibition of endothelial cells-mediated ATP release by spironolactone suppressed AAA formation and growth (2). Mechanistically, Ladd et al. revealed that the administration of spironolactone attenuated the release of extracellular adenosine triphosphate (ATP) from endothelial cells to mitigate the activation of macrophages, and smooth muscle cell-mediated remodeling. Several epidemiological observations have highlighted the possible role of diabetes mellitus in protection against AAA incidence and prevalence (3). In a review article, Picatoste et al. reviewed and summarized the available literature on the relationship between diabetes mellitus and AAA incidence and discussed the potential molecular pathways involved (4). Bontekoe and Liu offered a substantial review and assessment of the current literature utilizing Singel Cell RNA sequencing for the AAA inspection and discussed the upcoming usefulness of this technology (5).

Thoracic aortic aneurysms (TAA) are the second most common and life-threatening aortic disease. In an original article, Deng et al. established a novel model of proximal TAAs in mice by peri-adventitial elastase application in the proximal thoracic aorta via a midline incision in the anterior neck of the mice (6). The currently available elastase animal models of TAA are distal descending TAAs, limiting the knowledge of understanding proximal TAA pathologies. This new minimally invasive proximal TAA model avoids thoracotomy and tracheal intubation by elastase application in the peri-adventitia of the proximal thoracic aorta via a midline incision in the mouse on the anterior neck.

We appreciate all the authors of this Special Issue for their contributions. We hope the four articles published in this Special Issue will assist researchers in improving our understanding of the underlying molecular mechanisms of aortic aneurysms and developing therapeutic targets for aortic aneurysms.
